# Telemetric ICP monitoring in children: a national questionnaire-based study

**DOI:** 10.1007/s00381-024-06383-y

**Published:** 2024-04-08

**Authors:** Sarah Hornshøj Pedersen, Kasper Amund Henriksen, Sara Duus Gustafsen, Torben Skovbo Hansen, Rikke Guldager, Marianne Juhler

**Affiliations:** 1 Department of Neurosurgery, Copenhagen University Hospital, Rigshospitalet, Inge Lehmanns Vej 8, 2100 Copenhagen, Denmark; 2 Department of Neurosurgery, Aarhus University Hospital, Skejby, Palle Juul-Jensens Boulevard 99, 8200 Aarhus, Denmark

**Keywords:** Patient perception, Parental perception, Questionnaire, Intracranial pressure

## Abstract

**Purpose:**

Telemetric monitoring of intracranial pressure (ICP) facilitates long-term measurements and home monitoring, thus potentially reducing diagnostic imaging and acute hospital admissions in favour of outpatient appointments. Especially in paediatric patients, telemetric ICP monitoring requires a high level of collaboration and compliance from patients and parents. In this study, we aim to systematically investigate (1) patient and parent perception of telemetric ICP system utility and (2) hospital contact history and thus the potential cost-benefit of telemetric ICP monitoring in paediatric patients with a cerebrospinal fluid disorder.

**Methods:**

We conducted a nationwide questionnaire study, including paediatric patients with either a current or previous telemetric ICP sensor and their parents. Additionally, a retrospective review of electronic health records for all included children was performed.

**Results:**

We included 16 children (age range 3–16 years), with a total of 41 telemetric ICP sensors implanted. Following sensor implantation, the frequency of telephone contacts and outpatient visits increased. No corresponding decrease in hospital admissions or total length of stay was found. The telemetric ICP sensor provided most parents with an improved sense of security and was seen as a necessary and valuable tool in treatment guidance. The size and shape of the sensor itself were reported as disadvantages, while the external monitoring equipment was reported as easy to use but too large and heavy for a child to carry.

**Conclusion:**

Though, in quantitative terms, there was no cost-benefit of the telemetric ICP sensor, it contributed to extended parental involvement and a sense of improved safety.

**Supplementary Information:**

The online version contains supplementary material available at 10.1007/s00381-024-06383-y.

## Introduction

Implants for telemetric monitoring of intracranial pressure (ICP) have been available for more than a decade, and through the years, several technologies have been presented [1, 2]. Until recently, the most frequently used devices in European centres have been Raumedic Neurovent-P-tel (Raumedic AG, Helmbrechts, Germany) and Miethke Sensor Reservoir/MScio (Christoph Miethke GmbH & Co. KG, Potsdam, Germany). The Raumedic Neurovent-P-tel is, however, currently not commercially available.

Telemetric ICP monitoring facilitates measurements over a longer period, including home monitoring or monitoring in an outpatient setting. It is primarily used in patients with complex cerebrospinal fluid (CSF) disorders and may be of further value in treatment-guidance of paediatric patients unable to describe their symptoms. Previous reports focused on technical advantages and disadvantages in telemetric ICP monitoring [1–4], including complication rates [1, 3–7], implant-related costs [2, 7, 8], and potential cost reductions [7–9] related to the telemetric ICP sensor. In 2019, we reported our experience with a series of 20 children with a total of 32 telemetric ICP sensors implanted over a span of four years. In this period, three patients had their sensors removed at the patient’s or parents’ request, compared to eight removals or replacements due to technical defects and two due to minor skin complications [10].

A telemetric ICP sensor is a long-term implant that requires a high level of collaboration and compliance from both patients and parents. The documentation of family and patient perceptions of advantages vs. disadvantages is scarce, and though the Neurovent-P-tel previously has been described as unobtrusive, easy to use, and tolerable for the child by the families of four paediatric patients [8], the positive feedback may reflect a reduction in hospital admissions and diagnostic imaging following sensor implantation. As no further literature elaborating family and patient perceptions of telemetric ICP monitoring exists, this study is aimed at systematically investigating (1) patient and parent perceptions of system utility and (2) hospital contact history and thus the potential cost-benefit following implantation of a telemetric ICP sensor in a national paediatric patient cohort.

## Methods

In Denmark, children with a complex CSF disorder requiring long-term telemetric ICP monitoring are assessed and treated at the neurosurgical departments at either Copenhagen University Hospital or Aarhus University Hospital. Both neurosurgical departments have specialised hydrocephalus nurses guiding and monitoring patients, including managing external equipment for monitoring sessions during hospital admissions, measurements at the outpatient clinic, and home-monitoring sessions.

Through study advertisements and personal recruitment during scheduled hospital visits, all parents of children aged 0 to < 18 years with a current or previous telemetric ICP sensor in Denmark were invited to participate in the project. There were no additional inclusion criteria and no exclusion criteria.

### Design

In order to construct a comprehensive and adequate questionnaire, we used a qualitative approach with a focus group interview to extend our knowledge on the telemetric ICP sensor, including the parents’ verbal description [11]. This study was thus designed as a longitudinal study with (1) the focus group interview (data not shown), (2) a preliminary questionnaire, (3) a national questionnaire survey, and (4) a retrospective review of electronic health records prior to and following implantation of the telemetric ICP sensor.

#### Preliminary questionnaire

Based on clinical experience and the thematic data analysis of the focus group interview, four preliminary questionnaires adjusted to age were constructed: ‘Parents’, ‘Child age 4–8 years’, ‘Child age 9–12 years’, and ‘Child age 13–17 years’. To prevent pre-set answers, the questionnaires were validated by seven parents and their children. Amendments were discussed in single-based telephone interviews with the first author and subsequently added to the questionnaires. Lastly, detailed instructions for the child and parent were added, encouraging the parent to explain each question and help the child reflect on the answers.

#### National questionnaire survey

The parent questionnaire consisted of four main sections: demographics, measurements and treatment, patient and parent perceptions of system utility, and advantages and disadvantages. Questionnaires for the children were modified to examine the child’s perception of the abovementioned sections. Examples of questions can be found in Supplementary Table 1. The questionnaire was distributed among the participants in April and May 2020, and completed questionnaires were received between May and December 2020.

#### Retrospective review of electronic health records

Electronic health records were retrospectively reviewed after the completion of the questionnaires. Demographics for the child, information on implantation, management, and explantation of the telemetric ICP sensor, hospital contacts related to the CSF disorder, surgical history, and data on diagnostic imaging from two time periods were retrieved: ‘pre-implantation’, consisting of one year prior to implantation of their first telemetric ICP sensor, and ‘post-implantation’, consisting of the time with an implanted telemetric ICP sensor.

### Statistics

Data were stored in a REDCap database [12], and data analysis was conducted using R version 4.1.0 [13]. Quantitative data are presented as mean values with corresponding standard deviations. The post-implantation period and the counted events within it were adjusted to represent a one-year period, allowing direct comparisons with the pre-implantation period. Comparisons were performed twice, once involving all patients and once excluding two outliers (one from each department). Data from the two periods were compared using a two-tailed bootstrapping method for mean differences with 1000 resamples with replacement and are presented as mean differences with 95% CI.

### The telemetric ICP sensor and treatment strategy

The Neurovent-P-tel is a parenchymal sensor, while the Sensor Reservoir is connected to a ventricular catheter. Technical comparisons were previously published [2, 14]. Both sensors are activated by an external reader unit (Reader TDT1 readP (Neurovent-P-tel) or Antenna (Sensor Reservoir)) and connected through a cable to an external storage unit (Datalogger (Neurovent-P-tel) or Reader Unit Set (Sensor Reservoir)). Hence, both the Datalogger and the Reader Unit Set must be in close range of the child and carried during movements. The Reader-ring is rather small and can be held in place during movements or overnight measurements, whereas the Antenna is larger and thus can only be used for spot measurements or postural measurements, e.g., following a so-called manoeuvre protocol as suggested by Pennacchietti et al. [15].

### Ethics

The study was conducted with the Declaration of Helsinki [16] and registered with the Danish Data Protection Agency (P-2019-754). Parents provided written consent on behalf of their child and themselves to participate in the questionnaire study and the retrospective analysis. Participants were informed of the voluntary nature of the study and that withdrawal from the study was possible at any time with no implications. No participants withdrew from the study.

The producer of the Neurovent P-tel^®^ (Raumedic) guarantees that the device is electronically functional for 3 months, which determines their recommended implantation period. The Sensor Reservoir/M.Scio^®^ (Miethke), on the other hand, is constructed to allow integration into a shunt system with no recommended restriction in implantation duration. The manufacturer guarantees that the device has a life-long functionality. As there are no other safety concerns by not removing the telemetric implants, as removal requires additional surgery and as we are not required by Danish law to remove it, we decided from the beginning to leave the telemetric devices implanted unless a specific clinical need for explantation occurred either for patient safety reasons (e.g., infection, skin erosion and local pain) or because of the patient’s or the parents’ desire.

## Results

Nineteen children fulfilled the inclusion criteria, and 16/19 families accepted study participation. The included children represent a wide spectrum of hydrocephalus diagnoses (Table 1) and are geographically residing in all regions of Denmark. All patients had experience with the Neurovent-P-tel, while only one patient had a Sensor Reservoir.

**Table 1:** Distribution of diagnoses

**Diagnosis**		**Diagnoses in the cohort**
G80	Cerebral palsy	1
G91.0	Communicating hydrocephalus	3
G91.1	Obstructive hydrocephalus	4
G91.3	Post-traumatic hydrocephalus, unspecified	1
G91.8	Other hydrocephalus	2
G91.9	Hydrocephalus, unspecified	7
G93.2	Benign intracranial hypertension	4
G94.0	Hydrocephalus in infectious and parasitic diseases classified elsewhere	1
Q03.8	Other congenital hydrocephalus	4
Q03.8C	Internal congenital hydrocephalus	1
Q03.9	Congenital hydrocephalus, unspecified	1
Q07.0	Arnold-Chiari syndrome	2
Q75.0	Craniosynostosis	2

The included children represent a wide section of hydrocephalus diagnoses. Some children have more than one diagnosis, and thus the total number of diagnoses does not equal the number of children participating in the study

### National questionnaire survey

All parents and 11/16 children completed the questionnaire. Not completing the questionnaire was due to no recall of the telemetric ICP sensor/not being able to distinguish the telemetric ICP sensor from a shunt (*n* = 2), no language (*n* = 2), and age < 4 years old (*n* = 1). The included children (male = 62.5%) were ages 4–8 (*n* = 4), ages 9–12 (*n* = 4) and ages 13–17 (*n* = 3). Ten children had a telemetric ICP sensor at the time of the questionnaire, and nine had ICP monitored with the sensor within the last six months.

### Measurements and treatment

According to 93.8% of the parents, the telemetric ICP sensor partly or completely fulfilled its purpose, and only one parent regretted that the child had the telemetric ICP sensor implanted. Further, most parents answered that the sensor was a necessary tool (87.5%), and if needed, they would allow the implantation of a new sensor (62.5%). Children in age groups 9–12 and 13–17 were also asked if they wanted a new telemetric ICP sensor if needed; 28.6% said ‘yes’, 42.8% said ‘no’, and 28.6% did not respond. Nearly all parents (93.8%) saw the explantation strategy as an advantage.

Most parents reported a beneficial effect of the telemetric ICP sensor (75.0%) and stated that the sensor was a valued tool in treatment guidance (68.8%) and even ‘ensured’ or ‘partly ensured’ the right treatment (93.8%). As this beneficial effect could reflect a simultaneous reduction in hospital admissions and diagnostic and surgical procedures, the parents’ perceptions of this were elaborated. Regarding qualitative impressions following the implantation of the telemetric ICP sensor, 37.6% of the parents reported a reduction in hospital admissions, 68.8% reported fewer admission days, 75.0% reported fewer diagnostic examinations, and 56.3% reported fewer surgical procedures.

### Patient and parent perceptions of system utility

In general, the telemetric monitoring system was reported as easy to use (68.8%). However, 93.8% of the parents reported the size and weight of the external monitoring equipment as a disadvantage, while 62.5% reported the size and shape of the telemetric ICP sensor itself as a disadvantage. In relation to this, 81.3% of the parents agreed or partly agreed that the ICP sensor caused a cosmetic issue for the child. Half of the children aged 4 to 12 years, and all the children aged 13 to 17 years stated that the ICP measurements were painful. It was further reported by the majority of the children in all age groups that the ICP sensor itself caused pain or itched at times when ICP was not monitored (Table 2).

**Table 2:** Statements from the questionnaires

Parents						
**Statement**	**Agree**		**Partly agree**	**Disagree**	**Do not know**	
The Datalogger makes my child stand out	68.8		18.8	12.5	-	
The ICP sensor makes my child stand out	50.0		18.8	31.3	-	
The ICP sensor causes cosmetic issues	37.5		43.8	18.8	-	
Due to the ICP sensor, my child gets bullied	-		12.5	81.3	6.3	
My child is sad to have an ICP sensor	25.0		12.5	50.0	12.5	
My child is embarrassed to have an ICP sensor	12.5		12.5	75.0	-	
The ICP sensor itself causes skin irritation	43.8		6.3	37.5	12.5	
The ICP sensor itself causes pain	31.3		25.0	37.5	6.3	
ICP measurements are unpleasant for my child	56.3		12.5	31.3	-	
ICP measurements are painful for my child	50.0		18.8	31.3	-	
The ICP sensor makes me feel more secure	68.8		25.0	6.3	0.0	
The ICP sensor supports my parent-intuition	62.5		25.0	6.3	6.3	

Children age 4–8 years and age 9–12 years						
**Statement**	**Agree a lot**	**Agree**	**Both agree and disagree**	**Disagree**	**Disagree a lot**	**I do not recall**
I am glad to have an ICP sensor implanted	25	-	50	-	25	-
	25	-	25	-	50	-
I am sad to have an ICP sensor implanted	25	-	50	-	25	-
	25^a^	-	25^a^	25^a^	-	-
The ICP sensor itself causes pain	25	50	-	-	25	-
	25	-	25	-	50	-
The ICP sensor itself itches	75	-	-	-	25	-
	50	25	-	-	25	-
ICP measurements are painful	25	25	50	-	-	-
	25	25	-	-	25	25
Other children tease me with the ICP sensor	-	-	50	-	50	-
	-	-	25	-	75	-

Children age 9–12 years						
**Statement**	**Agree a lot**	**Agree**	**Both agree and disagree**	**Disagree**	**Disagree a lot**	**I do not recall**
I am thinking of the ICP sensor every day	25	25	25	-	25	-
The ICP sensor makes me feel different than other children	25	-	-	-	75	-
It is easy to conduct ICP measurements outside my home e.g. in school	-	25	-	-	75	-

Children age 13–17 years						
**Statement**	**Agree a lot**	**Agree**		**Disagree**	**Do not know**	**I do not recall**
I am glad to have an ICP sensor implanted	-	33.3		33.3	33.3	-
I am sad to have an ICP sensor implanted	-	-		66.6	33.3	-
The ICP sensor itself causes pain	33.3	66.6		-	-	-
The ICP sensor itself itches	-	-		100	-	-
ICP measurements are painful	-	100		-	-	-
Other children tease me with the ICP sensor	-	-		100	-	-
I am thinking of the ICP sensor every day	-	-		100	-	-
The ICP sensor makes me feel different than other children	33.3	33.3		33.3	-	-

	**Yes easily**	**Yes, but it is difficult**		**No**	**I do not recall**	
I can do what I normally do when I conduct an ICP measurement	-	33.3		66.6	-	

The parents and children were asked whether they agreed with different statements. Statements were adjusted to the child’s age. Numbers are listed in percent

^a^Except one statement, all had a response rate of 100%

A telemetric ICP sensor facilitates home monitoring of the ICP, which was reported as an advantage by 93.8% of parents. In theory, home-monitoring should reflect the child’s everyday life more accurately than in-hospital measurements; however, only 68.8% stated that home-monitoring sessions ‘highly’ or ‘to some degree’ reflected the child’s everyday life. More statements on system utility and patients’ and parents’ perceptions of it are shown in Table 2.

### Retrospective review of electronic health records

The 16 included children had a total of 41 telemetric ICP sensors implanted throughout the study period (median 2, IQR 1–3). Six children had one sensor, while ten children had the sensor replaced either once (*n* = 5) or more than once (*n* = 5). Reasons for implantation were either diagnostic (*n* = 2) or assessment of treatment effect (shunt, endoscopic third ventriculostomy or Diamox; *n* = 39) (Table 3). Nine out of 16 children had an implanted sensor when the electronic health records were reviewed, as one sensor was removed after conducting the questionnaire and prior to the electronic health records review.

**Table 3:** Explantation of the telemetric ICP sensor

**Reason**	**Number of ICP sensors removed/replaced**
Patient’s or parent’s request	1
Local pain/irritation around the sensor	4
Minor wound defect	1
Liquor accumulation around the sensor	1
Infection	2
No longer functional^a^	17
Unknown	6

A total of 41 ICP sensors were implanted, and 32 ICP sensors were either removed or replaced with a new ICP sensor

^a^One Raumedic Neurovent-P-tel was replaced with a Miethke Sensor Reservoir

On average, the telemetric ICP sensor was functional for 337.6 days, ranging from 12 to 1290 days (median 244 days, IQR 122–485 days), and was used for an average of 13.1 home measurements and 13.9 in-hospital-measurements per child (range 1–55 and 1–88, respectively).

Between the pre-implantation and post-implantation periods adjusted to represent one year, telephone contacts and outpatient contacts with the hydrocephalus nurse increased from 1.19 to 6.61 calls per year and from 0.19 to 3.25 visits per year, respectively. The same trend was seen for consultations with the neurosurgeon, with an increase from 2.25 to 3.22 calls per year and an increase from 4.56 to 6.40 visits per year.

Neither hospital admissions nor days admitted to the hospital changed after the implantation of the telemetric ICP sensor. Looking at surgical procedures post-implantation, fewer conventional cable-based ICP measurements were performed, while the number of external ventricular drains was similar in both periods. The number of shunt procedures, however, increased (estimated mean difference including two outliers at 0.81 [95% CI 0.06 to 1.68], estimated mean difference excluding two outliers at 0.68 [95% CI − 0.12 to 1.56]). The same trend, though not significant, was seen for the number of endoscopic third ventriculostomies (Table 4). Lastly, the frequency of MRI and X-rays was comparable in the pre-implantation period and the post-implantation period (Table 4), while the number of CT scans was reduced (estimated mean difference including two outliers at − 0.67 [95% CI − 1.56 to 0.38], estimated mean difference excluding two outliers at − 1.17 [95% CI − 1.87 to − 0.34]). Excluding the two outliers did not affect the remaining analyses, and the data are thus not shown.

**Table 4:** Number of events pre-implantation vs. post-implantation

Number of events			**Pre-implantation** (Mean (SD))	**Post-implantation** (Mean (SD))	**Estimated mean diff** (95% CI)
**Hospital contacts**	Hydrocephalus nurse	Telephone	1.19 (1.38)	6.61 (4.06)	*5.42 (3.48 to 7.22)*
		Outpatient clinic	0.19 (0.54)	3.25 (3.33)	*3.07 (1.52 to 4.69)*
	Neurosurgeon	Telephone	2.25 (2.52)	3.22 (2.79)	*0.99 (0.12 to 2.04)*
		Outpatient clinic	4.56 (3.61)	6.40 (4.98)	*1.83 (0.01 to 3.90)*
**Admissions**	Number of hospital admissions		3.50 (1.83)	4.48 (3.42)	0.96 (− 0.67 to 2.70)
	Days of admission		12.75 (10.46)	13.82 (17.81)	1.02 (− 6.35 to 8.66)
**Surgical history**	EVD		0.13 (0.34)	0.17 (0.37)	0.05 (− 0.22 to 0.30)
	Shunt surgery		1.31 (1.20)	2.13 (2.04)	*0.81 (0.06* to *1.68)*
	ETV		0.00 (0.00)	0.24 (0.64)	0.24 (0 to 0.55)
**Diagnostic examination/evaluation**	Cerebral imaging	X-ray	1.19 (1.33)	1.09 (1.93)	− 0.09 (− 1.01 to 0.81)
		CT	2.31 (0.95)	1.64 (2.04)	− 0.67 (− 1.56 to 0.38)
		MRI	1.13 (1.26)	1.02 (0.84)	− 0.10 (− 0.93 to 0.70)
	ICP measurement	Shunt chamber	0.00 (0.00)	0.18 (0.41)	0.18 (0 to 0.39)
		Cable-based	0.44 (0.51)	0.02 (0.07)	* − 0.42 (− 0.67 to − 0.19)*
		Telemetric (in hospital)	NA	5.3 (5.2)	NA
		Telemetric (at home)	NA	5.9 (3.5)	NA

The table shows retrieved events one year up to implantation of the telemetric ICP sensor (pre-implantation) and compared to events with a telemetric ICP sensor (post-implantation). The number of events within the post-implantation period was adjusted to represent a one-year period. Values in italic indicate a significant change in number of events

X-ray of the cranium, thorax, and abdomen to visualise shunt placement/shunt series radiographs including skull radiographs

*ICP* intracranial pressure, *EVD* external ventricular drainage, *ETV* endoscopic third ventriculostomy, *CT* computed tomography scan, *MRI* magnetic resonance imaging

## Discussion

This is the first study systematically investigating patient and parent perceptions of telemetric ICP monitoring in paediatric patients. We report that parents of children with a telemetric ICP sensor in general report a beneficial effect, in which the ICP sensor ensures or partly ensures correct treatment. Contrary to our expectations and findings in previous publications [7, 8], the implantation of a telemetric ICP sensor did not reduce the number of hospital admissions or admission days. However, we believe that parental involvement in disease management and treatment decisions contributes to a sense of improved safety, as also indicated by Tschan et al. [9]. The opportunity to monitor and regulate treatment seems to lead to a lower threshold for hospital contacts and thus an increase in telephone and outpatient contacts, which have also previously been reported by Bjornson et al. [7].

### Advantages and disadvantages

Measurement of ICP in the patient’s home theoretically reflects the child’s everyday life and thus provides a more accurate long-term picture of ICP during normal daily activities than in-hospital measurements. However, in our cohort, only two-thirds of the parents stated that a home monitoring session in fact reflected or partly reflected the child’s everyday life, as the children were physically limited by the size and weight of external monitoring equipment and by the need for skin-fixation of the reader-ring. The external monitoring equipment from both Raumedic and Miethke has previously been reported as a disadvantage [4, 14], and a new and smaller design has therefore also recently been launched from Raumedic (RAUMED Home ICP: size 140 × 72 × 16 mm and weight 200 gramme).

Parents in our cohort agree with previous positive statements in terms of system utility [4, 8]. However, they disagree in terms of tolerable measurements, and nearly 80% of the parents stated that ICP measurements are unpleasant or even painful for the child. This was supported by most of the children, regardless of age. The cosmetic disadvantage found in our cohort has previously been briefly reported for the Miethke Sensor Reservoir [14].

### A heterogeneous patient population

Apart from two implantations for diagnostic purposes, our study population includes a selected cohort of children with complex CSF disorders. While previous studies report an overall clinical improvement and positive impact on shunt management following implantation of a telemetric ICP sensor [3, 7, 17], the frequency of hospital admissions in our cohort was unchanged, and the clinical complexity seemed to persist. It is possible that this could be due to the progression of other factors than ICP regulation, e.g., medication-induced headaches or psychological stress, which should be recognised and referred for appropriate treatment. Further, a child with a CSF disorder might adapt to a high ICP, and the clinical management will therefore change due to the ICP readings and not due to the child’s symptoms.

Though our cohort represents most hydrocephalus-related diagnoses and children aged 3 to 16 years, the data do not reflect children with less complicated CSF disorders. A case series of 4 patients with a Neurovent P-tel [8] reported an overall cost reduction comparing the number of pre-implantation events and post-implantation events. However, 2/4 had more inpatient stays in the post-implantation period, and in 1/4, the number of imaging studies was increased. In only 1/4, the need for inpatient stays, shunt series/skull radiographs, and MRIs was completely removed in the post-implantation period. Although we found no overall reduction in hospital admissions, we did find a reduction in CTs. Excluding the two outliers in our cohort, the reduction was significant, emphasising an individual effect of telemetric ICP monitoring and the variability expected from small case series. It is thus not unlikely that an individual patient analysis would show a decrease in hospitalisation and in diagnostic procedures in some patients. Further, Barber et al. [8] evaluated patient-related costs from the initial presentation of the CSF disorder and prior to publication of results, whereas we evaluated clinical events one year prior to implantation and adjusted events following implantation to one year.

### Perspectives

The data in this study show no overall cost-benefit of the telemetric ICP sensor. On the contrary, the implantation of the sensor was followed by a significant increase in both telephone contacts and visits in the outpatient clinic, and thus an increase in use of hospital resources. The ICP sensor did, however, contribute to parental involvement in disease management and thus a sense of improved safety.

Future development of telemetric ICP monitoring equipment for use in paediatric patients should take several things into consideration; (1) size, weight and method for fixation of the external equipment; (2) unlimited, natural movement range; (3) home-monitoring sessions should reflect the child’s everyday life; and (4) freedom from discomfort or pain during ICP monitoring sessions or related to the ICP sensor itself.

### Supplementary Information

Below is the link to the electronic supplementary material.

Supplementary file1 (DOCX 89 KB)

## Data Availability

Data can be provided upon request.

## Declarations

### Disclaimer

The funder had no role in study design, in collection, analysis, and interpretation of data; writing of the report; or the decision to submit the article for publication.
