# Supplementary material for: Telemetric ICP monitoring in children: a national questionnaire-based study
Source: Childs Nerv Syst. 2024 Apr 8;40(8):2391–9. doi: 10.1007/s00381-024-06383-y (PMC11269325; doi:10.1007/s00381-024-06383-y)
Supplement: Supplementary file 1 — Supplementary file1 (DOCX 89 KB) [file 381_2024_6383_MOESM1_ESM.docx]

# Supplementary Information

## Supplementary Table 1 Examples of questions/statements from the four questionnaires

For children age 4-8 years and age 9-12 years the answers were supplemented with emojis;

**Agree a lot Disagree**

**Agree Disagree a lot**

**Both agree and disagree I do not recall**

|  | **Question/statement and options** |
| --- | --- |
| **Parents** | Does ICP measurements in-home reflect your child’s everyday-life?  *Highly; To some degree; Lowly; Not at all, Do not recall* |
|  | Did the telemetric ICP sensor fulfill its purpose?  *Yes; Partly; No; I do not know* |
|  | Specify whether the size and weight of the Datalogger is  *an Advantage; a Disadvantage; Neither an advantage or a disadvantage; I have not thought about it* |
|  | How often do you communicate with the hydrocephalus nurse?  *<1/week; 1/week; 1-3/month; 1-3/6 months; 1-3/year; It varies; Do not recall* |
| **Children**  **4-8 years** | I am glad to have an ICP sensor implanted  *Agree a lot; Agree; Both agree and disagree; Disagree; Disagree a lot; I do not recall* |
|  | I am sad to have an ICP sensor implanted  *Agree a lot; Agree; Both agree and disagree; Disagree; Disagree a lot; I do not recall* |
|  | ICP measurements are painful  *Agree a lot; Agree; Both agree and disagree; Disagree; Disagree a lot; I do not recall* |
|  | Other children tease me with the ICP sensor  *Agree a lot; Agree; Both agree and disagree; Disagree; Disagree a lot; I do not recall* |
| **Children**  **9-12 years** | I am thinking of the ICP sensor every day  *Agree a lot; Agree; Both agree and disagree; Disagree; Disagree a lot; I do not recall* |
|  | The ICP sensor makes me feel different than other children  *Agree a lot; Agree; Both agree and disagree; Disagree; Disagree a lot; I do not recall* |
|  | It is easy to conduct ICP measurements outside my home e.g. in school  *Agree a lot; Agree; Both agree and disagree; Disagree; Disagree a lot; I do not recall* |
|  | If the neurosurgeon suggests it, will you have a new ICP sensor?  *Yes; No* |
| **Children**  **13-17 years** | I am thinking of the ICP sensor every day  *Agree a lot; Agree; Disagree; I do not know; I do not recall* |
|  | The ICP sensor makes me feel different than other children  *Agree a lot; Agree; Disagree; I do not know; I do not recall* |
|  | I can do what I normally do when I conduct an ICP measurement  *Yes easily; Yes but it is difficult; No; I do not recall* |
|  | If the neurosurgeon suggests it, will you have a new ICP sensor?  *Yes; No* |
